# Anabolic therapy for osteoporosis: update on efficacy and safety

**DOI:** 10.20945/2359-3997000000566

**Published:** 2022-11-11

**Authors:** Leonardo Bandeira, E. Michael Lewiecki

**Affiliations:** 1 Universidade Federal de São Paulo São Paulo SP Brasil Universidade Federal de São Paulo, São Paulo, SP, Brasil; 2 Grupo Fleury Recife PE Brasil Grupo Fleury, Recife, PE, Brasil; 3 New Mexico Clinical Research & Osteoporosis Center Albuquerque NM USA New Mexico Clinical Research & Osteoporosis Center, Albuquerque, NM, USA

**Keywords:** Osteoporosis, treatment, teriparatide, abaloparatide, romosozumab

## Abstract

Anabolic agents for the treatment of osteoporosis increase bone density, improve bone strength, and reduce fracture risk. They are distinguished from antiresorptive drugs by their property of increasing osteoblastic bone formation. Teriparatide and abaloparatide are parathyroid hormone receptor agonists that increase bone remodeling with bone formation increasing more than bone resorption. Romosozumab is a humanized monoclonal antibody to sclerostin that has a “dual effect” of increasing bone formation while decreasing bone resorption. The bone forming effects of anabolic therapy appear to be self-limited, making it imperative that it be followed by antiresorptive therapy to enhance or consolidate the beneficial effects achieved. Teriparatide, abaloparatide, and romosozumab each have unique pharmacological properties that must be appreciated when using them to treat patients at high risk for fracture. Clinical trials have shown a favorable balance of expected benefits and possible risks. Anabolic therapy is superior to bisphosphonates for high-risk patients, with greater benefit when initial treatment is with an anabolic agent followed by an antiresorptive drug, rather than the reverse sequence of therapy. Recent clinical practice guidelines have included recommendations with examples of patients who are candidates with anabolic therapy.

## INTRODUCTION

Osteoporosis is a systemic skeletal disease characterized by an imbalance of bone remodeling with bone resorption in excess of bone formation, leading to bone loss, degradation of bone microarchitecture, and high risk of fractures (1). Because of “coupling” of bone resorption and formation, osteoclastic bone resorption and osteoblastic bone formation generally, but not always, move in the same direction, and not always with the same magnitude. In young adults, the rate of bone turnover is moderate, with resorption approximately the same as formation, resulting is stability of bone density. However, women with postmenopausal osteoporosis typically have a high rate of bone turnover, with bone resorption greater than bone formation (2). In aging men, an increase in bone turnover is associated with bone loss (3). Younger women and men with idiopathic osteoporosis may have normal or slightly increased bone resorption with low bone formation due to osteoblast dysfunction (4,5). With glucocorticoid-induced osteoporosis (GIO), there is an increase in bone resorption and a decrease in bone formation (6). Chronic kidney disease mineral and bone disorder with renal osteodystrophy is composed of a broad range of skeletal disorders, including osteomalacia, sometimes co-existing with osteoporosis, with bone turnover rates ranging from high to low (7). Regardless of the pattern of disrupted bone remodeling, the eventual consequence is likely to be reduction of bone strength and increased risk of fractures.

All approved drugs for the treatment of osteoporosis modulate bone remodeling in ways that increase bone mineral density (BMD), strengthen bones, and reduce the risk of fractures. Antiresorptive (anti-remodeling) drugs (e.g., bisphosphonates, denosumab) reduce the rate of bone remodeling, inhibiting activity of osteoclasts more than osteoblasts, resulting in fewer and smaller bone remodeling units (8). This allows for greater filling of the remodeling space, increased secondary mineralization of bone, and an increase in BMD, more with long-term denosumab that with bisphosphonates. Bone structure is preserved with bisphosphonates, while denosumab has been associated with improvement in cortical bone structure, possibly due to preservation of modeling-based bone formation while inhibiting bone resorption (9).

Osteoanabolic compounds are defined by their ability to stimulate bone formation. The formation of new bone can restore, at least partially, degraded bone microarchitecture that is characteristic of osteoporosis and an independent predictor of fracture risk (10). This bone forming effect cannot be achieved by antiresorptive drugs that decrease bone formation as well as bone resorption. The three osteoanabolic compounds currently in clinical use (Table 1) can be classified as parathyroid hormone (PTH) receptor agonists (teriparatide [Forteo^®^, Eli Lilly and Company, Indianapolis, IN, USA] and abaloparatide [Tymlos^®^, Radius Health Inc, Boston, MA, USA]) and anti-sclerostin therapy (romosozumab [Evenity^®^, Amgen Inc., Thousand Oaks, CA, USA]). Teriparatide (PTH [1-34]) is a synthetic peptide containing the first 34 amino acids of endogenous human PTH. Parathyroid hormone-related protein (PTHrP) is a protein with homology to PTH at the amino terminus. Abaloparatide (PTHrP [1-34]) is a synthetic analog of PTHrP. It is identical to endogenous human PTHrP through the first 22 residues but thereafter includes multiple amino acid substitutions that influence its interactions at the receptor level. Abaloparatide was designed to retain potent anabolic activity with less bone resorption, less calcium-mobilizing potential, and improved room temperature stability compared with teriparatide. Romosozumab is a humanized monoclonal antibody to sclerostin, a glycoprotein produced primarily by osteocytes that blocks the canonical Wnt signaling bone formation pathway. By binding to sclerostin, romosozumab allows the engagement of Wnt ligands with their co-receptors, low-density lipoprotein receptor-related protein 5 and 6 (LRP5/6) and Frizzled, resulting in a “dual-effect” of stimulating bone formation while reducing bone resorption (11). This is different than the effects of the PTH receptor agonists that stimulate bone formation and resorption. There is evidence that the PTH receptor agonists primarily stimulate remodeling-based bone formation (i.e., filling and overfilling of bone remodeling units) while bone formation with sclerostin inhibition in primarily modeling based (i.e., de novo bone formation that is independent of bone remodeling units) (12). These differences in the mechanisms of action of the anabolic agents contribute to differences in the therapeutic profiles and adverse effects. This review is an update on what is known about the efficacy and safety of teriparatide, abaloparatide, and romosozumab.

**Table 1:** Summary of selected properties of interest for three approved anabolic compounds for treatment of osteoporosis

Property	Teriparatide	Abaloparatide	Romosozumab
Regulatory approval	2002	2017	2019
Molecule	PTH(1–34)	PTHrP(1–34)	Humanized Monoclonal Antibody
Mechanism	PTH receptor agonist	PTH receptor agonist	Anti-sclerostin
Bone formation	Increases	Increases	Increases
Bone resorption	Increases	Increases	Decreases
Dose	20 mcg SC daily	80 mcg SC daily	210 mg SC monthly
Administration	Self-injection	Self-injection	By Healthcare Professional
Duration limit	24 months*	24 months lifetime	12 months (may repeat)
Indications	Postmenopausal osteoporosis Male osteoporosis GIO	Postmenopausal osteoporosis	Postmenopausal osteoporosis
Rat osteosarcoma	Yes	Yes	No
Warning to avoid in patients at high risk for osteosarcoma	Yes	Yes	No
Warning to avoid in patients with myocardial infarction or stroke in the past year	No	No	Yes

* Branded teriparatide (Forteo) for more than 2 years during a patient's lifetime should only be considered if a patient remains at or has returned to having a high risk for fracture.

PTH: parathyroid hormone; PTHrP: PTH related protein; SC: subcutaneous; GIO: glucocorticoid induced osteoporosis.

## EFFICACY

### Teriparatide

The idea of using a PTH analog as a bone-forming agent seems paradoxical since it is known that PTH is a hormone that primarily stimulates bone resorption, as observed in patients with primary hyperparathyroidism. However, 40 years ago it was observed that intermittent administration of PTH induced an anabolic effect on the bone, predominantly at trabecular skeletal sites (13).

The mechanism of action of the PTH agonists is not completely elucidated, but it seems there are several participating signaling pathways that initially stimulate osteoblasts and later osteoclasts (14–17). Thus, after teriparatide administration, an early increase in bone formation markers is observed, with a peak between 6-12 months, followed by an increase in bone resorption markers to a lesser extent than the formation. Thus, an “anabolic window” is generated, with a predominance of formation over resorption, especially in the first months of treatment (18,19).

The approval of teriparatide came out after the study by Neer and cols. which showed a BMD gain, especially in the lumbar spine (LS), and a reduction in the risk of fractures in patients who used the medication. In this study, 1,637 postmenopausal women with previous vertebral fractures received teriparatide or placebo for 18 months. An increase of 9.7% and 13.7% in the lumbar spine BMD was observed with doses of 20 and 40 mcg/day, respectively, compared with placebo (p < 0.001 for both). The teriparatide group had a 2.8% and 2.6% increase of femoral neck (FN) BMD, and 5.1% and 3.6% in the total hip (TH) BMD for 20 and 40 mcg/day dosages, respectively, compared with placebo (p < 0.001 for all) (20).

The study showed a risk reduction of 65% and 69% for vertebral fractures in the groups receiving 20 and 40 mcg, respectively, compared with the placebo group (p < 0.001 for both). The reduction in non-vertebral fractures was 53% and 54% for 20 and 40 mcg/day groups, respectively (p < 0.05 compared with placebo for both) (20). Another more recent randomized controlled trial (RCT), the VERtebral fracture treatment comparisons in Osteoporotic women (VERO) study, compared teriparatide with risedronate in 1,360 postmenopausal women with low BMD and a history of fractures. After 24 months, new vertebral fractures occurred in 5.4% of patients on teriparatide and 12% of patients on risedronate (p < 0.0001). Clinical fractures occurred in 4.8% and 9.8% of patients using teriparatide and risedronate, respectively (p = 0.0009) (21).

Teriparatide is also effective in glucocorticoid-induced osteoporosis, as observed in studies by Saag and cols., who first evaluated 428 men and women aged 22 to 89 years with low bone mass who were using glucocorticoids. Patients were randomized to receive teriparatide 20 mcg or alendronate 10 mg daily. After 18 months, a significantly greater BMD increase was observed in the teriparatide group compared with the alendronate group at the LS (7.2% *vs.* 3.4%, respectively, p < 0.001) and TH (3.8% *vs.* 2.4%, respectively, p < 0.005). The teriparatide group had fewer vertebral fractures than the alendronate group (0.6% *vs.* 6.1%, respectively, p = 0.004), with no difference in non-vertebral fractures (5.6% *vs.* 3.7%, respectively, p = 0.36) (22).

An extension of this study evaluated patients for an additional 18 months. After a total treatment duration of 36 months, the differences between groups remained, with more BMD gains in the teriparatide group than the alendronate group (11% *vs.* 5.3%, respectively, at the LS; 5.2% *vs.* 2.7%, respectively, at the TH, p < 0.001 for both). At the femoral neck, which is a predominantly cortical skeletal site where teriparatide might have lesser BMD gains, there was a greater increase in BMD with teriparatide than with alendronate (6.3% *vs.* 3.4%, p < 0.001). The superiority of teriparatide *vs.* alendronate for preventing vertebral fractures remained at the end of 36 months (1.7% *vs.* 7.7%, respectively, p = 0.007), while there was no difference in the incidence of non-vertebral fractures (teriparatide 7.5 % *vs.* 7% alendronate, p = 0.84) (23).

In a study with 437 men with low bone mass, teriparatide, at doses of 20 and 40 mcg/day, was compared with placebo. After 11 months, the LS BMD gain was superior in the teriparatide groups (40 mcg 9% *vs.* 20 mcg 5.9% *vs.* placebo 0.5%, p < 0.001 for all comparisons). The FN BMD increased by 2.9% in the 40 mcg group, 1.5% in the 20 mcg group, and 0.3% in the placebo group (p < 0.03 for all comparisons) (24).

Once a course of therapy with teriparatide is completed, the patient must be transitioned to an antiresorptive agent, without which there will be rapid BMD loss. Switching to denosumab or bisphosphonates after teriparatide is followed by further BMD gains (25–27).

### Abaloparatide

The efficacy of abaloparatide was evaluated in the phase 3 Abaloparatide Comparator Trial in Vertebral Endpoints (ACTIVE), which compared 80 mcg/day of the medication with 20 mcg/day of teriparatide and placebo in 1,901 postmenopausal women with osteoporosis and very high fracture risk. After 18 months, the abaloparatide and teriparatide groups had fewer vertebral fractures compared with the placebo group (abaloparatide *vs.* placebo – *hazard ratio* [HR] 0.14, teriparatide *vs.* placebo – HR 0.2, p < 0.001 for both). Moreover, the abaloparatide group had fewer major osteoporotic fractures (MOF) compared with the placebo group (HR 0.3, p < 0.001) and teriparatide group (HR 0.45, p = 0.03). Regarding nonvertebral and clinical fractures, the abaloparatide group had a lower incidence of events than the placebo group (HR 0.57, p = 0.049 for nonvertebral fractures; HR 0.57, p = 0.02 for clinical fractures), but there were no between-group differences in the comparisons abaloparatide *vs.* teriparatide and teriparatide *vs.* placebo (28).

In the ACTIVE, the abaloparatide group had greater BMD gains than the placebo group at all skeletal sites. At the end of 18 months, the abaloparatide group had almost 12% BMD gains at the LS and approximately 4% BMD gains at the hip regions of interest compared with the placebo group (p < 0.001 for all). Compared with teriparatide, there was no statistical difference between groups for BMD change at the LS, but abaloparatide was superior at the TH and FN (p < 0.001 for both) (28).

As with teriparatide, abaloparatide generates an initial increase in bone formation with a subsequent increase in bone resorption. The PTHrP analog leads to an elevation in bone formation markers, with an approximately 80% peak in the first month and a subsequent progressive decline, but remaining approximately 30% above baseline after 18 months. This increase in bone formation is lower than that observed with teriparatide, which reaches more than 120% increase in the sixth month, remaining above 100% compared to the baseline until the end of the 18 months. However, lower increase in bone formation with abaloparatide is offset by a lesser increase in bone resorption than teriparatide (abaloparatide leads to a C-terminal telopeptide – CTX – peak of less than 20% in the third month, returning to baseline before 18 months, while teriparatide leads to an approximately 50% CTX peak, remaining above 20% from baseline at month 18) (28).

The use of abaloparatide should also be followed by an antiresorptive drug. The transition from abaloparatide to bisphosphonate was assessed in an extension of ACTIVE (ACTIVExtend). Patients who completed the first study with 18 months of abaloparatide (n = 558) and placebo (n = 581) were eligible to receive alendronate for up to 24 months. In the total treatment period of 43 months from ACTIVE baseline to the end of ACTIVExtend, the abaloparatide/alendronate group had an 84% lower risk of new vertebral fractures compared with the placebo/alendronate group (incidence 0.9% *vs.* 5.6%, p < 0.001). This was approximately the difference reached at the end of ACTIVE and was therefore maintained with alendronate. The abaloparatide/alendronate group also had 39% lower risk of nonvertebral fractures (p = 0.038), 34% lower risk of clinical fractures (p = 0.045), and 50% lower risk of MOF (p = 0.011) compared to the placebo/alendronate group (29).

The BMD gains achieved in the abaloparatide group in ACTIVE were further enhanced with alendronate, reaching greater than 14% at the LS, 6% at the TH, and 5% at the FN at the end of ACTIVExtend (p < 0.001 compared with the placebo/alendronate for all) (29).

### Romosozumab

The romosozumab pivotal fracture trial was the Fracture Study in Postmenopausal Women with Osteoporosis (FRAME), which enrolled 7,180 postmenopausal women with osteoporosis, randomized into romosozumab or placebo groups for 1 year. After this period, both groups switched to open-label denosumab for 1 more year. A study extension followed patients for an additional 12 months on denosumab. At the end of the first year, BMD increased by 13.3% at the LS, 6.8% at the TH, and 5.2% at the FN in the romosozumab group (*vs.* 0.0, 0.0, and −0.7%, respectively, in the placebo group; p < 0.001 for all). At the end of the third year in the FRAME Extension, the group that received romosozumab for 12 months followed by open-label denosumab for 24 months achieved a BMD increase of 18.1% at the LS, 9.4% at the TH, and 8.2% at the FN compared with 7.5%, 5.2%, and 4.8%, respectively, in the group receiving placebo for 12 months followed by open-label denosumab for 24 months (p < 0.001 for all) (30,31).

Another phase 3 trial, the Active-Controlled Fracture Study in Postmenopausal Women with Osteoporosis at High Risk (ARCH), compared the efficacy of romosozumab with alendronate in 4,093 postmenopausal women with osteoporosis and a previous fracture. The patients were randomized to receive romosozumab or alendronate in the first year, followed by open-label alendronate for both groups for the following 24 months. After the first 12 months of study drug, BMD gains in the romosozumab group were similar to those seen with romosozumab in FRAME and significantly greater compared with alendronate (13.7% *vs.* 5% at the LS, 6.2% *vs.* 2.8% at the TH, 4.9% *vs.* 1.7% at the FN, respectively; p < 0.001 for all). Alendronate after 12 months of romosozumab maintained the BMD increases achieved with romosozumab, which is a different pattern than with romosozumab followed by denosumab in FRAME, where there were further increases in BMD (32).

The Study to Evaluate the Effect of Treatment With RomosozUmab Compared with Teriparatide in PostmenopaUsal Women at High Risk of Fracture Previously Treated with a BisphosphonatE (STRUCTURE) compared the effects of romosozumab with another osteoanabolic agent, teriparatide, in 436 postmenopausal women who had previously been treated with oral bisphosphonates. After 12 months, the romosozumab group had greater BMD gains than teriparatide at all sites (7.2% *vs.* 3.5% at the LS, 2.3% *vs.* −0.8% at the TH, and 2.1% *vs.* −1.1% at the FN, respectively; p < 0.0001 for all) (33).

The dynamic of bone markers summarizes the “dual-effect” of the anti-sclerostin antibody, that is, stimulation of bone formation together with suppression of bone resorption. An increase in P1NP (amino-terminal procollagen type 1) is observed, with a peak before the third month, reaching more than 100% increase, followed by a gradual reduction in the following months. CTX falls by about 50% in the first month, remaining reduced after this period (30,32,33).

The anabolic window generated by romosozumab results in BMD gains that are associated with a reduction in fracture risk, the main desired outcome for an osteoporosis medication. In FRAME and the FRAME Extension, a 73% risk reduction in vertebral fractures was observed after 12 months compared with placebo (p < 0.001). The risk reduction was maintained after the transition to denosumab. After 36 months, the risk of vertebral fractures was 66% lower in the group receiving romosozumab in the first year compared with the group receiving placebo in the first year (in years 2 and 3, both groups used denosumab). The same pattern was observed for clinical fractures, with romosozumab reducing the risk by 36% at the end of the first year and the romosozumab-denosumab group reducing the risk by 27% at the end of the third year (p = 0.008 and 0.004, respectively, compared with placebo for 1 year and placebo-denosumab groups) (30,31).

In FRAME, there was no significant difference in the risk of non-vertebral fractures between the romosozumab-denosumab and placebo-denosumab groups at the end of 24 months. However, in the FRAME Extension, after continuing denosumab for an additional 12 months, there was a statistically significant 21% decrease in the risk of non-vertebral fractures in the group receiving romosozumab in the 12 months *vs.* placebo in the first 12 months (p < 0.05). The finding of no reduction of non-vertebral fracture risk in the first 24 months with romosozumab-denosumab has been attributed to the large proportion of study subjects in Latin America, for whom there was a lower baseline risk of fractures. Excluding Latin American participants from the analysis, the risk reduction of romosozumab compared with placebo increased from 25% to 42% in the first 12 months (p = 0.04) (30).

In addition to the risk reduction of vertebral, clinical and non-vertebral fractures after 36 months, the group that used romosozumab in the first year had a reduction in the risk of major non-vertebral (RRR = 27%, p = 0.01), major osteoporotic (RRR = 30%, p = 0.006), new vertebral (RRR = 65%, p < 0.001) and multiple vertebral (RRR = 90%, p < 0.001) fractures compared to the group that took a placebo in the first year (31).

Romosozumab is more effective than alendronate for preventing fractures. The anti-sclerostin antibody led to a 37% reduction in the risk of vertebral fractures after one year compared with alendronate (p = 0.003). After transitioning to alendronate, fracture risk continued to decline. The group that used romosozumab in the first year followed by alendronate had a 48% reduction in the risk of vertebral fractures after the second year compared with the group receiving alendronate since the beginning (p < 0.001) (32). Romosozumab for 1 year followed by alendronate was also more effective than alendronate for the whole period in reducing clinical (RRR = 27%, p < 0.001), non-vertebral (RRR = 19%, p < 0.001) and hip fractures (RRR = 38%, p = 0.02), after the second year (32).

As with other osteoanabolic agents, romosozumab should be followed by an antiresorptive medication to consolidate and enhance the benefits achieved, as its discontinuation without switching reduces BMD to levels near to pre-treatment in approximately 1 year (34).

Romosozumab has also been studied in men. In the phase 3 placeBo-contRolled study evaluatIng the efficacy anD safety of romosozumab in treatinG mEn with osteoporosis (BRIDGE), 245 men aged 55 to 90 years with osteoporosis received romosozumab or a placebo. The romosozumab group had significantly greater BMD gains compared with the placebo group after 12 months (LS 12.1% *vs.* 1.2%; TH 2.5% *vs.* −0.5%; FN 2.2% *vs.* −0.2%, respectively; p < 0.001 for all) (35).

Table 2 summarizes the clinical efficacy of the osteoanabolic agents.

**Table 2:** Summary of the clinical efficacy of the approved osteoanabolic agents (based on outcomes of phase 3 studies)

**Teriparatide**	Postmenopausal women More BMD gains *vs.* placebo at all skeletal sites Risk reduction of vertebral and nonvertebral fractures compared with placebo Risk reduction of vertebral and clinical fractures compared with risedronate GIO Greater BMD gains *vs.* alendronate at all skeletal sites Risk reduction of vertebral fractures compared with alendronate Men Greater BMD gains *vs.* placebo at LS and FN
**Abaloparatide**	Postmenopausal women Greater BMD gains *vs.* placebo at all skeletal sites Greater BMD gains vs. teriparatide at TH and FN Risk reduction of vertebral, major osteoporotic, nonvertebral and clinical fractures compared with placebo Risk reduction of MOF compared with teriparatide Risk reduction of vertebral, clinical, nonvertebral and major osteoporotic fractures in an abaloparatide followed by alendronate group compared with placebo followed by alendronate group
**Romosozumab**	Postmenopausal women Greater BMD gains *vs.* placebo at all skeletal sites Greater BMD gains *vs.* alendronate at all skeletal sites Greater BMD gains *vs.* teriparatide at all skeletal sites Risk reduction of vertebral, clinical and nonvertebral* fractures compared with placebo Risk reduction of vertebral and clinical fractures compared with alendronate Risk reduction of vertebral, clinical, nonvertebral, major nonvertebral and major osteoporotic fractures in a romosozumab followed by denosumab group compared with placebo followed by denosumab group Risk reduction of vertebral, clinical, nonvertebral and hip fractures in a romosozumab followed by alendronate group compared with an alendronate group Men Greater BMD gains *vs.* placebo at all skeletal sites

* Excluding Latin America patients, which had a lower fracture risk.

BMD: bone mineral density; LS: lumbar spine; TH: total hip; FN: femoral neck; MOF: major osteoporotic fracture; GIO: glucocorticoid induced osteoporosis.

### Safety

#### Teriparatide

In the pivotal fracture trial that led to the regulatory approval in 2002, teriparatide was generally well tolerated, with no significant differences among the three groups (20 mcg subcutaneously [SC] daily, 40 mcg SC daily, and placebo SC daily) in the number of deaths and hospitalizations, cardiovascular disorders, urolithiasis, or gout (20). For the approved 20 mcg dose of teriparatide, dizziness (9%) and leg cramps (6%) were more common than with placebo (6% and 1%, respectively); mild hypercalcemia (serum calcium > 10.6 mg/dL 4-6 hours after dosing) occurred at least once in 11% *vs.* 2% with placebo; mean 24-hour urinary calcium increased by 30 mg, with no subjects having hypercalciuria (> 300 mg per day); and mean serum uric acid increased by 13-20%. There were no reported cases of atypical femur fracture or osteonecrosis of the jaw. There was no increase in the incidence of cancer and no cases of osteosarcoma.

Concern about a potential increase in the risk of osteosarcoma was raised because of preclinical studies in Fischer 344 rats showing that prolonged high doses of teriparatide were associated with the development of osteosarcoma (36). When these findings were released, the pivotal fracture trial for teriparatide was suspended earlier than planned and never resumed, with study participants receiving teriparatide for an average of 19 months with median observation time 21 of months (20). Subsequently, because of a favorable benefit *vs.* risk profile, teriparatide received regulatory approval for the treatment of women with postmenopausal osteoporosis at high risk for fracture, with a boxed warning that it should not be prescribed for patients who are at increased baseline risk for osteosarcoma, including those with Paget's disease of bone or unexplained elevations of alkaline phosphatase, open epiphyses, or prior external beam or implant radiation therapy involving the skeleton.

In the years since its approval, there has been no evidence for an increase in the risk of osteosarcoma in humans treated with teriparatide (37). In 2020, 18 years after initial approval, the brand name product label was changed, eliminating the boxed warning, demoting the statement about osteosarcoma to “Warnings and Precautions,” and changing the 2-year restriction to read that it may be used for more than 2 years “if a patient remains at or has returned to having a high risk for fracture” (38). Limitations of teriparatide include the requirement for refrigeration, the need for daily self-injections, and high cost. Biosimilar teriparatide products are now available.

#### Abaloparatide

In the pivotal fracture trial (ACTIVE) with abaloparatide 80 mcg SC daily, open-label teriparatide 20 mcg SC daily, and placebo SC daily, there were no evident differences between treatment groups in overall treatment-emergent adverse events, serious adverse events, or deaths (28). There were more adverse events leading to study discontinuation in the abaloparatide (9.9%) group than with teriparatide (6.8%), or placebo (6.1%). The most common adverse events leading to study discontinuation with abaloparatide were nausea (1.6%), dizziness (1.2%), headache (1.2%), and palpitations (0.9%). Hypercalcemia, defined as serum albumin-corrected calcium > 10.6 mg/dL, was less common with abaloparatide (3.4%) than with teriparatide (6.4%). Hypercalciuria was similar with abaloparatide (11.3%) and teriparatide (12.5%). There were no cases of atypical femur fracture, osteonecrosis of the jaw, or osteosarcoma.

As with teriparatide, preclinical studies of abaloparatide in Fischer 344 rats found a dose-dependent increase in development of osteosarcoma (39). When abaloparatide received regulatory approval in 2017 for the treatment of women with postmenopausal osteoporosis at high risk for fracture, there was a boxed warning regarding potential risk of osteosarcoma and a restriction to 2 years of lifetime use. At the time of this writing, the boxed warning is still in place. Limitations of abaloparatide include daily self-injection and high cost. The delivery device does not have to be refrigerated after first use.

#### Romosozumab

In the double-blind one-year period of the pivotal fracture trial (FRAME) with romosozumab 210 mg SC once monthly *vs.* placebo SQ once monthly, the incidence of adverse events and serious adverse events was balanced between the two groups (30). The incidence of death, cancer, osteoarthritis, and adjudicated serious cardiovascular events and deaths was similar. Injection site reactions were more common with romosozumab (5.2%) than with placebo (2.9%). There was one case (<0.01%) of atypical femur fracture 3.5 months after starting romosozumab *vs.* none with placebo in a patient who reported prodromal pain at the site of the fracture prior to study enrollment. There was one case (<0.01%) of osteonecrosis of the jaw after 12 months of romosozumab *vs.* none with placebo in a patient with ill-fitting dentures. The median albumin-corrected serum calcium level was lower at 1 month in the romosozumab group compared with placebo (median change from baseline −2.2% *vs.* 0.0%, respectively), with one patient (<0.01%) in the romosozumab having hypocalcemia. There were no cases of osteosarcoma.

Concern regarding a potential risk of cardiovascular disease was raised in another clinical study (ARCH) with an initial one-year double blind period with participants receiving monthly romosozumab 210 mg SC or weekly alendronate 70 mg orally (32). Positively adjudicated serious cardiovascular adverse events were observed more often with romosozumab (2.5%) than with alendronate (1.6%). Despite reviews of the data, it remains unclear whether this numerical difference was due to romosozumab increasing the risk, alendronate decreasing the risk, or chance alone (40). Due to an abundance of caution, the product label has a boxed warning that it may increase the risk of myocardial infarction, stroke, and cardiovascular death, and that is should not be used in patients who have had a myocardial infarction or stroke within the preceding year (41).

## DISCUSSION

In recent years, osteoporosis clinical practice guidelines developed by organizations such as the American Association of Clinical Endocrinologists (AACE) (42), the Endocrine Society (ES) (43,44), the Bone Health and Osteoporosis Foundation (BHOF) (45), the International Osteoporosis Foundation (IOF) and European Society for Clinical and Economic Aspects of Osteoporosis and Osteoarthritis (ESCEO) (46), and the National Osteoporosis Guideline Group (NOGG) (47) have identified a new category of “very high fracture risk.” The US-based organizations (AACE, ES, BHOF) offer examples of very high risk, such as having very low T-score (<-3.0), multiple fractures, or recent fractures. Guidelines from the UK (NOGG) and Europe (IOF, ESCEO) have proposed that very high risk be defined as probability of major osteoporotic fracture and/or hip fracture that is above the pre-existing age-adjusted intervention level, using a range of multipliers, with FRAX with or without inclusion of femoral neck BMD, suggesting that this identifies about 10% of women over age 50 years as being at very high risk (47). Regardless of the methodology used to define very high risk, there is a universal theme that initial therapy with an anabolic agent should be considered in such patients.

The rationale for starting with anabolic therapy is founded on randomized head-to-head clinical trials showing a more rapid and greater reduction of fracture risk with anabolic drugs compared with antiresorptive drugs in high-risk patients (21,22,32,48) and recognition of the importance of treatment sequence, with a greater BMD response with anabolic therapy followed by antiresorptive therapy rather than antiresorptive therapy followed an anabolic therapy (49,50). While any treatment for osteoporosis is better than none, there are clear advantages to initiating treatment with an anabolic agent in appropriately selected patients. Limitations of anabolic therapy include inconvenience of dosing (daily self-injections with teriparatide and abaloparatide; monthly injections by a healthcare professional with romosozumab), lack of availability in some regions, and high cost.

Discontinuation of osteoporosis therapy is followed by a decline of BMD (51). This is especially problematic after discontinuation of long-term treatment with denosumab, which results in a rapid decreased in BMD, rise and overshoot of bone turnover markers, and increase of fracture risk (52). BMD declines slowly after stopping bisphosphonates, giving rise to the concept of a bisphosphonate “holiday” (intentional temporary withholding of drug administration) (53). BMD rapidly decreases after stopping teriparatide (54–56), although there is observational evidence for persistence of anti-fracture benefit for as long as 18 months for vertebral fractures (55) and as long a 30 months for non-vertebral fractures (54). In a phase 2 study of romosozumab with a small number of participants, there was a large decrease of BMD after discontinuation with no reports of vertebral fractures within 24 months of transitioning from romosozumab to placebo (34). We are not aware of any studies of abaloparatide discontinuation. Since osteoporosis is considered to be a lifelong disease (57), and the skeletal effects of treatment diminish, sooner or later, after treatment is stopped, transitions of therapeutic agents must be considered. It is the standard of care to follow anabolic therapy with an antiresorptive agent (42,43).

In conclusion, for patients at very high risk of fracture, especially those who have one or more prior fractures, the ideal initial therapy is with an anabolic agent. It is essential to follow this with an antiresorptive medication to consolidate and enhance the anabolic effects. Treatment decisions should consider all available clinical information, including patient preference.
